# A review on the utility of sensitivity analysis in trauma research: Methods and challenges from recent trauma studies

**DOI:** 10.1007/s00068-025-03034-y

**Published:** 2025-12-18

**Authors:** Ayman El-Menyar, Ahammed Mekkodathil, Hassan Al-Thani, Brad H. Pollock

**Affiliations:** 1 Clinical Research Trauma Surgery, Hamad Medical Corporation, Doha, Qatar; 2 Clinical Medicine, Weill Cornell Medicine, Doha, Qatar; 3 Trauma & Vascular Surgery Section, Hamad Medical Corporation , Doha, Qatar; 4 Department of Public Health Sciences, School of Medicine, University of California Davis, Davis, CA US

**Keywords:** Clinical trials, Missing data, Robustness, Sensitivity analysis, Trauma research

## Abstract

**Background:**

In trauma research, the complexity of patient presentations, variability across clinical environments, and diversity in outcomes often introduce substantial uncertainty into the interpretation of findings. Sensitivity analysis (SA) is a vital methodological tool for examining the stability and reliability of research conclusions by testing how they respond to changes in analytical methods, model structures, unmeasured confounding, and foundational assumptions.

**Methods:**

A literature review was conducted to provide an overview of current knowledge and updates on the application of sensitivity analysis in trauma research. A search was performed in PubMed and Google Scholar for studies conducted in humans and published in English between May 2000 and May 2025.

**Results:**

This review explored the crucial role that SA plays in trauma studies, outlining key techniques, including addressing missing data through multiple or single imputation, evaluating protocol noncompliance, adjusting for baseline imbalances, validating statistical assumptions, and conducting subgroup analyses. These strategies are particularly relevant in trauma research, where data fragmentation, wide population variability, and ethical challenges are common. Drawing on landmark trauma studies including PROMPTT, PROPPR, PATCH-Trauma, PAMPer, and recent neural network applications, we demonstrated how SA can be utilized to evaluate model accuracy, determine variable importance, and assess the consistency of treatment effects.

**Conclusion:**

Although the implementation of SA in trauma research remains uneven across the field, establishing SA as a standard element of study design and reporting is vital. Doing so not only bolsters the transparency and trustworthiness of analytical results but also enhances the reproducibility and applicability of findings, ultimately supporting more robust and clinically meaningful advancements in trauma care.

## Background

Trauma research is uniquely challenging due to the inherent complexity of patient presentations, the variability across clinical settings, and the vast heterogeneity in outcomes. Together, these factors introduce substantial uncertainty, making it challenging to draw conclusions that are both robust and generalizable. Sensitivity analysis (SA) is a vital methodological tool to address this challenge. By systematically examining how study results respond to variations in analytical methods, model specifications, unmeasured variables, or key assumptions, SA provides a framework for evaluating the reliability of research findings [1–4]. At its core, SA aims to identify which outcomes are most impacted by uncertain or unsupported assumptions, thereby signaling where findings may be fragile or highly contingent on specific analytical decisions [1–6].

Clinical trials, including those conducted in trauma settings, are typically built on several assumptions, whether related to randomization, distributional properties of study variables, or fidelity to treatment adherence. When these assumptions are violated, the validity of the study’s conclusions can be compromised [7, 8]. In such instances, SA is essential for assessing how these deviations influence the results and whether the core conclusions remain intact [7, 9]. A close alignment between the outcomes of primary analyses and those of corresponding sensitivity tests significantly bolsters confidence in the study’s conclusions [7, 10]. Still, interpreting consistency across analyses requires careful consideration of the research question, context, and the broader implications of the findings [10–12].

Evaluating the robustness of study outcomes is a fundamental component of responsible research, particularly when results could be affected by analytical choices or external factors [7, 8]. As such, SA should be regarded as a mandatory part of any comprehensive analysis plan [7, 9]. Leading regulatory agencies reinforce this viewpoint. The U.S. Food and Drug Administration (FDA) and the European Medicines Agency (EMA) emphasize the necessity of SA in their guidelines on statistical principles, highlighting that trial conclusions must be tested for their sensitivity to data limitations, underlying assumptions, and modeling strategies [7, 8]. Similarly, the National Institute for Health and Care Excellence (NICE) in the United Kingdom recommends the use of SA to explore alternative scenarios and quantify uncertainty, particularly in economic evaluations [10]. Given the fast-paced, resource-limited, and often unpredictable nature of trauma care, rigorous, transparent SA is essential to ensure the evidence generated is both trustworthy and clinically meaningful. This review is the first of its kind to explore the role of SA in clinical and trauma research, outlining key methodological approaches, highlighting real-world applications from major trauma studies, and identifying challenges and recommendations for broader, more impactful implementation.

A literature review was conducted to provide an overview of current knowledge and updates on the application of sensitivity analysis in trauma research. A search was performed in PubMed and Google Scholar for studies conducted in humans and published in English between May 2000 and May 2025. The search keywords included “sensitivity analysis” AND “trauma”. Only studies with full text availability or informative abstracts were considered. Titles and abstracts were screened to identify studies that discussed the methods, applications, or implications of sensitivity analysis in trauma-related research, including clinical trials, observational studies, and secondary analyses. The key terms used for the search included “Sensitivity analysis” AND “Trauma.” As there were no studies primarily aimed at addressing this review topic, the study quality assessment was not applicable. We thoroughly examined the methods sections of the screened studies to ensure they were relevant to the objectives of our review. Reference lists of key articles were also reviewed to identify additional pertinent publications.

## Use of Sensitivity Analysis in Clinical and Health Research

SA plays a pivotal role in studies assessing treatment effects, particularly in addressing the distortions introduced by biases such as unmeasured confounding, model misspecification, and missing data. These issues can significantly compromise the validity of study conclusions. Yet, despite widespread acknowledgment of its importance, SA remains underutilized in clinical and health research. In an evaluation of 135 articles from major medical and health economics journals, Thabane et al. [1] found that only 27% included any form of sensitivity analysis. The disparity was more pronounced in medical journals, where only 20% reported SA, compared to 31% in health economics publications. Even among randomized controlled trials (RCTs), just 17% incorporated SA, revealing a persistent disconnect between methodological guidance and actual research practice.

The necessity of SA is even more pronounced in observational studies, especially those utilizing routinely collected healthcare data (RCD), such as electronic health records and insurance claims. These real-world data sources offer valuable insights into treatment effects. Still, they are highly susceptible to bias, misclassification, unmeasured confounding, immortal-time bias, and ever-evolving treatment exposures, all of which threaten the study’s internal validity. In this context, SA serves as a tool to quantify the extent to which such biases might alter the study’s conclusions and tests whether results hold under different analytical assumptions. Researchers frequently apply SA to explore alternative definitions of key variables (e.g., exposure, outcomes, or covariates), modify cohort inclusion/exclusion criteria, or test different modeling approaches, such as competing risk models or marginal structural models that address time-varying confounding (e.g., time-dependent covariates), or E-values to estimate the strength of unmeasured confounding required to overturn observed associations.

A meta-epidemiological study by Xu et al. [13], which reviewed 256 observational studies using routinely collected healthcare data (RCD) between 2018 and 2020, further underscored these issues. While 59.4% of studies conducted at least one SA, more than 40% failed to perform any. Among those who did, the median number of analyses was three, with the most common focus on alternative definitions (65.1%), modeling strategies (55.9%), and study design variations (48.7%). Importantly, inconsistencies between primary and sensitivity analyses were common. Of the 131 studies that reported comparative results, over half (54.2%) showed significant variation in effect estimates across at least one SA. On average, the effect size differed by 24%, and in nearly 20% of cases, the direction of the estimate reversed. In 14.5% of studies, the confidence intervals from sensitivity analyses did not overlap with those from the primary analysis, raising serious concerns about the robustness of the findings. Yet these discrepancies were often overlooked, with only 12.6% of studies meaningfully addressing their potential implications.

Several factors influence both the likelihood of conducting sensitivity analyses and the consistency of results. Studies published in high-impact journals (Q1) or those with pre-registered protocols were more likely to report SA. At the same time, studies that conducted more than three SA scenarios were more likely to uncover inconsistencies. In contrast, research reporting larger effect sizes, using active comparators, or appearing in high-impact journals tended to yield more consistent results across analyses. These patterns suggest that both methodological discipline and editorial expectations shape the adoption and interpretation of SA.

Despite its clear value, SA remains unevenly applied and frequently under-interpreted. Relying solely on primary analyses without thoroughly exploring potential sources of bias can lead to overconfident or misleading conclusions. To promote greater transparency and rigor, researchers are encouraged to systematically identify plausible sources of bias, select and justify suitable SA methods (such as E-values to assess unmeasured confounding or negative control analyses), report findings clearly, and critically interpret any inconsistencies. In observational research, where randomization is absent and confounding cannot be fully controlled, SA should no longer be treated as an optional supplement but rather as a core element of study design, analysis, and interpretation.

## Sensitivity Analysis Methods

Sensitivity analysis includes a suite of methodological tools used to assess the robustness of study findings by varying assumptions, model structures, or analytic approaches. In trauma and clinical research, where missing data, heterogeneity, and protocol deviations are common, SA may be crucial for assessing whether conclusions remain valid under various scenarios. Below are the key strategies employed in SA, along with implementation and presentation approaches.

### a) Handling Outliers

Outliers may disproportionately influence regression estimates and distort conclusions. SA commonly involves comparing results with and without outliers or using robust regression, which down-weights extreme values. If conclusions remain consistent, findings are considered robust. For example, in a weight change study, regression coefficients varied slightly across methods, but all approaches supported the main conclusions [5].

### b) Addressing Missing Data

Missing data is pervasive in clinical research. SA strategies include:

- **Complete-case analysis**, which excludes incomplete observations, may reduce power and bias results if data are not Missing Completely at Random (MCAR) [3].
- **Single imputation** methods, such as mean substitution, regression-based filling, or last observation carried forward (LOCF), are simple but tend to underestimate variability.
- **Multiple imputation (MI)** generates various datasets that incorporate the uncertainty inherent in missingness, producing more reliable and unbiased estimates [1, 5].

Comparing outcomes across these methods allows researchers to judge the robustness of their findings relative to missing data assumptions.

### c) Evaluating Non-Compliance or Protocol Deviations

In randomized controlled trials (RCTs), **intention-to-treat (ITT)** analysis is a standard approach that preserves the original randomization in the analyses. ITT analyses may then be compared with:

- **Per-protocol (PP)** analyses (including only fully adherent participants),
- **As-treated (AT)** analyses (classifying participants by actual treatment received irrespective of how participants were randomly assigned to treatment groups) [1].
- Consistency of conclusions across ITT, PP, and AT estimates reinforces the robustness of results, while divergence may reveal the influence of protocol deviations.

### d) Using Different Outcome Definitions

Outcomes may vary in how they are defined, by cutoff thresholds, diagnostic codes, or timing (e.g., 24-hour vs. 30-day mortality). SA tests how altering these definitions impacts results, which is particularly important in observational studies with flexible coding systems or administrative data [1, 5].

### f) Addressing Competing Risks

When outcomes like death preclude the occurrence of other events (e.g., readmission), SA uses competing-risks models, such as **Fine & Gray** or **Lunn-McNeill**, to test whether ignoring these risks biases the conclusions [1].

## g) Inadequate Adjustment for Baseline Imbalance

### h) Testing Statistical Model Assumptions

SA often compares results using:

- **Different distributional assumptions** (e.g., normal distribution vs. t-distribution),
- **Model types** (e.g., Poisson vs. negative binomial),
- **Parametric vs. non-parametric methods**, or.
- **Bayesian vs. frequentist approaches** [1, 5, 12].

Transformations (e.g., log-transforms, splines) may be applied to variables that make them more normally distributed. For semi-continuous variables, such as coronary artery calcium (CAC), two-part models are often employed [12]. More advanced approaches include instrumental variable analysis or a high-dimensional propensity score to assess the impact of potential unmeasured confounding [12].

### i) Varying Exposure or Covariate Definitions

In observational studies, exposure classification (e.g., duration or time since exposure) or covariate definitions may vary. SA tests whether results change when using alternative definitions [12], which is especially relevant in studies with medication adherence variability.

### j) Using Alternative Comparison Groups

Sensitivity can be tested by including multiple comparator groups (e.g., recent vs. former users, active vs. passive comparators) to assess whether the observed effect is specific to one comparison and not due to confounding by indication or other biases [12]. This is only possible if the comparison groups are collected in exactly the same way (even in parallel designs).

### k) Replication Across Data Sources

Applying the same analysis across independent datasets or time periods is an SA method for testing generalizability. Results that hold across data sources are more likely to be externally valid and not dataset-specific [12].

### l) Assessing Selection Bias

When there is potential selection bias (e.g., due to differential loss to follow-up or differential consent rates), SA can simulate various bias scenarios (e.g., worst-case and best-case assumptions) to estimate how much bias would be needed to nullify findings [12].

### m) Examining Subpopulations

SA can assess the consistency of treatment effects across subgroups such as age groups, sex, race/ethnicity, comorbid conditions, or trauma type (e.g., blunt vs. penetrating). This improves understanding of potential effect modification and overall generalizability of the findings [12].

### n) Using Alternative Cohort Definitions

Changing inclusion or exclusion criteria (e.g., age limits, injury severity) allows for testing whether the primary results are robust to how the study cohort is defined, an important consideration in trauma studies with diverse case mixes [12].

## Implementation Approaches

### a) Spreadsheet-Based Sensitivity Analysis

Simple spreadsheet tools can be used for “what-if” analyses or visualizing variation in assumptions. Methods such as 3D array plots or “rule-out” sensitivity plots help estimate the level of confounding required to overturn observed associations [12].

### b) Statistical Software-Based approaches

Software packages like R, SAS, and Stata can perform advanced sensitivity analyses, including:

- Multiple imputation,
- GEE and multilevel models,
- Competing risk regression,
- Propensity score adjustment,
- Instrumental variable methods [12].

## c) Presentation of Results

### d) SA Findings can be Reported in:

- **Narrative form** to describe methodological impact,
- **Tabular summaries** comparing estimates across scenarios,
- **Graphical outputs** (e.g., tornado plots, imputation bands) that illustrate the sensitivity of results to key assumptions or variable definitions [12].

## Use of Sensitivity Analysis in Trauma Research

Despite its acknowledged utility, SA remains underused and often underreported within clinical research, especially in observational studies where it is frequently omitted or inadequately interpreted [1, 3, 13]. Nevertheless, its application in trauma research is steadily growing, driven by the distinct methodological, operational, and ethical challenges characteristic of the field [14–16].

### Heterogeneity in Injury Mechanisms and Trauma Experience

Trauma populations are inherently heterogeneous, with injuries arising from diverse mechanisms such as blunt force, penetrating trauma, burns, or polytrauma. These differences influence injury patterns, physiological responses, treatment timelines, and outcomes. Furthermore, individuals’ subjective experiences of trauma, shaped by factors such as gender, early life adversity, and cumulative exposures, may affect symptom reporting, care-seeking behavior, and participation in research. Such heterogeneity can introduce inconsistencies in data interpretation and complicated subgroup analyses. SA enables researchers to test whether conclusions are consistent across trauma types and patient subgroups, enhancing generalizability and interpretability [14, 15, 17].

### Dynamic and Fragmented Data Environments

Trauma care often occurs in fast-paced, high-pressure clinical settings with time constraints and limited resources. As a result, data collection may be incomplete, abbreviated, or inconsistently recorded. Additionally, variability in electronic health record (EHR) systems, limited data interoperability between treatment centers, and inconsistent documentation standards contribute to data fragmentation [18, 19]. Many trauma registries fail to capture the full continuum of care, from prehospital interventions through rehabilitation and long-term outcomes. In this context, SA is crucial for assessing how varying assumptions, definitions, or data harmonization approaches impact study results.

### Missing or Incomplete Data

Missing data are pervasive in trauma research due to the chaotic nature of emergencies, patient incapacitation, and system limitations. Key physiological or treatment variables may be missing completely at random (MCAR), missing at random (MAR), or missing not at random (MNAR), often with complex patterns that are difficult to detect [14, 24–26]. SA provides structured approaches to examine the impact of different methods for handling missing data, including complete-case analysis, single imputation, and multiple imputations. Comparing results across these methods helps determine whether findings are robust or particularly sensitive to the chosen approach.

### Population Heterogeneity and Ethical Complexities

Trauma populations often include vulnerable groups, such as individuals with mental illness, substance use disorders, those with unstable housing, those with prior violence exposure, pr those who face unique barriers to research participation. Ethical challenges, including informed consent in acute settings, the risk of re-traumatization, and the selective exclusion of high-risk patients, may introduce selection bias and reduce external validity [20–23]. SA can test whether including or excluding specific subgroups changes study conclusions, reinforcing both scientific rigor and ethical integrity.

### Model Specification and Analytical Assumptions

Trauma studies frequently rely on complex modeling frameworks that require assumptions about data structure, variable distributions, and predictor-outcome relationships. Trauma data often violates these assumptions due to non-normality or nonlinear effects. SA enables exploration of robustness across alternative model specifications, such as different functional forms, variable categorizations, and non-parametric methods. For example, a secondary analysis of the PAMPer trial showed how reclassifying continuous variables (e.g., plasma age) into different thresholds can test sensitivity to categorization choices [28]. Similarly, machine learning analyses apply SA by tuning models and altering architectures to validate findings [15].

### Variable Importance and Predictor Robustness

SA can also evaluate the relative importance of predictor variables, especially in clinical prediction models or artificial intelligence applications. In trauma care, where rapid triage and decision-making are vital, models that use fewer but highly informative predictors are advantageous. Neural network analyses have introduced SA methods that systematically test the effect of removing or substituting variables on model performance. These techniques can identify redundancy, challenge assumptions about standard variables (e.g., respiratory rate, gender), and guide the development of more efficient prediction tools [15].

### External Validation and Replication Across Datasets

Although sometimes overlooked as SA, applying models or algorithms to independent datasets, whether from different populations, centers, or time periods, serves as a form of robustness assessment. External validation, as demonstrated in the PROMMTT study’s use of pre-existing massive transfusion models on its dataset, helps confirm whether results generalize across clinical contexts [24]. This is especially relevant in trauma, where injury patterns and care practices vary widely across regions and institutions.

In summary, SA plays a multifaceted and essential role in trauma research. It strengthens the credibility of statistical findings and improves transparency, reproducibility, ethical soundness, and interpretability. Incorporating SA into study design, analysis, and reporting is critical to ensuring that trauma research generates reliable, clinically actionable insights, particularly given the challenging, data-limited, and heterogeneous environments typical of trauma care.

## Examples of Sensitivity Analysis in Trauma Studies

Despite its recognized importance, SA remains underutilized and underreported in clinical research, particularly in observational studies, where it is often omitted or inadequately interpreted [1, 3, 13]. Nevertheless, its use in trauma research is growing, reflecting the field’s unique methodological, operational, and ethical complexities [14–16]. The following examples from major trauma studies demonstrate how various types of SA are employed to enhance the robustness, credibility, and generalizability of findings in this challenging research context.

### a) Prehospital Plasma During Air Medical Transport Trial (PAMPer Trial)

This multicenter, cluster-randomized, phase 3 superiority trial [27] was designed to determine the efficacy and safety of prehospital administration of thawed plasma (TP) in injured patients at risk for hemorrhagic shock compared to standard-care resuscitation during air medical transport. The trial involved 501 patients transported by air medical personnel from 27 air medical bases to 9 trauma centers. Of these, 230 patients were assigned to the plasma group, and 271 to the standard-care group.

In the primary analysis, the researchers found that 30-day mortality was significantly lower in the plasma group (23.2%) than in the standard-care group (33.0%). To account for missing vital status data for 20 patients (10 in each group), the primary analysis utilized multiple imputation. Researchers assess the robustness of the findings by conducting sensitivity analyses for 30-day mortality, excluding all patients with unknown vital status at 30 days in one analysis, then assuming all patients with unknown vital status at 30 days survived in another analysis, and assuming a 50% mortality rate for patients with unknown vital status at 30 days in a third analysis. The results consistently showed that significant differences in 30-day mortality persisted between the plasma and standard-care groups across all sensitivity analyses, indicating that the observed benefit of prehospital plasma was robust and not dependent on assumptions about missing outcome data.

### b) Plasma Age Sensitivity in Pamper Trial

This study was a secondary analysis of the PAMPer trial, which had previously established the benefit of prehospital plasma in reducing 30-day mortality among trauma patients at risk of hemorrhagic shock [28]. The purpose of this secondary analysis was to explore whether the duration of plasma thawing, up to its 5-day validity period, was associated with differences in clinical outcomes or biomarker expression.

For the primary analysis, plasma was categorized into two groups based on the median thawed time: “YOUNG” plasma (thawed for 0–1 days) and “OLD” plasma (thawed for 2–5 days). The overall findings indicated that both the YOUNG and OLD plasma groups had lower 30-day mortality than standard care, consistent with the original PAMPer trial results. Additionally, plasma age itself did not significantly affect mortality when comparing YOUNG versus OLD plasma.

To further test the stability of these findings, a sensitivity analysis was performed to examine the effect of plasma at its most extreme thawed age. This involved comparing the clinical outcomes of the “YOUNG” plasma group (plasma thawed for 0–1 days) with those of the “EXTREME OLD” plasma subgroup (plasma thawed for 3–5 days). This specific comparison allowed the researchers to confirm if plasma administered very close to its expiration date (within the 5-day validity period) yielded different results in patient survival compared to fresher plasma.

The sensitivity analysis showed that TP age did not significantly affect 30-day mortality, even at these extreme durations (3–5 days). When directly comparing YOUNG plasma to EXTREME OLD plasma, there was no statistically significant difference in 30-day mortality observed [HR 0.89 (95% CI 0.47 to 1.49), *p* = 0.72]. This finding was crucial, as it supported the safety and efficacy of TP use throughout its permitted 5-day shelf life in clinical practice, despite prior animal and ex vivo studies suggesting potential degradation of plasma components with increasing storage time.

###  c) Association of Prehospital Plasma with Distinct Biomarker Expression (PAMPer Trial)

This study is a secondary, *post hoc* analysis of data from the PAMPer trial, which aimed to explore the biological mechanisms through which prehospital plasma improves survival in severely injured trauma patients, as demonstrated in the original trial [29]. The central hypothesis was that prehospital plasma would reduce immune dysregulation and endothelial damage.

Blood samples were collected from a subset of PAMPer trial patients; some were unavailable due to urgent interventions or early death. While these unsampled patients had similar ISS scores, they showed higher early mortality rates. Although overall biomarker levels did not differ significantly between the plasma and standard care groups, survivors exhibited distinct immune and endothelial profiles. Hierarchical clustering analysis (HCA) identified two patient clusters (A and B) with distinct injury patterns and biomarker expression profiles. Cluster A, marked by more severe injuries, showed a 30-day survival benefit from prehospital plasma. Among patients with ISS > 30, plasma administration was associated with lower levels of proinflammatory and endothelial damage markers, and higher levels of reparative immune mediators. These findings suggest that prehospital plasma modulates immune responses and reduces endothelial injury, particularly in the most critically injured patients.

A generalized linear model (GLM) was used as a sensitivity analysis to test the robustness of these associations in the most severely injured subgroup. This focused approach assessed whether the biological effects of plasma persisted under more severe injury. The model was adjusted for key clinical confounders, including ISS, GCS, prehospital hypotension, fluid resuscitation variables, and INR, thereby minimizing the risk of confounding bias from baseline differences across trial arms.

GLM results showed that by 24 h post-admission, prehospital plasma was significantly associated with lower levels of proinflammatory mediators and endothelial damage markers, as well as higher levels of reparative immune mediators. These findings support the hypothesis that plasma attenuates inflammation and endothelial injury, reinforcing the biological plausibility of its observed survival benefit in the original trial.

##  d) Cost-Effectiveness of Prehospital Plasma Transfusion in Unstable Trauma Patients

This secondary analysis of the PAMPer Trial evaluated the cost-effectiveness of prehospital transfusion of thawed plasma (TP) compared with standard-care fluid resuscitation in trauma patients with hemorrhagic shock during air medical transport [30]. A decision tree, stratified by injury mechanism (blunt vs. penetrating trauma), and a Markov model with a 1-year cycle and a 120-year time horizon for survivors beyond 30 days were used to assess costs and outcomes. The model incorporated prehospital transfusion, in-patient and lifetime healthcare costs, as well as quality of life measures. TP was found to be cost-effective, yielding 6.71 quality-adjusted life years (QALYs). Over 90% of the incremental costs were attributed to in-hospital (26%) and post-discharge care (68.8%), reflecting improved survival in the TP group. Additionally, TP patients required fewer transfusions, resulting in an average cost saving of $891.77.

To verify the robustness of these findings, the study conducted comprehensive sensitivity analyses to test key assumptions in the decision tree and Markov model. These included one-way (varying one input at a time), two-way (varying two inputs simultaneously), and Monte Carlo probabilistic sensitivity analyses (varying multiple inputs across realistic ranges). Variables such as patient age, number of air transport bases, annual trauma volume, and blunt trauma mortality rates were adjusted to determine whether the conclusions would remain consistent. The results demonstrated that TP remained cost-effective across a wide range of scenarios. Notably, the Monte Carlo analysis showed that TP was preferred in over 81% of simulations at the $100,000/QALY threshold, underscoring the robustness of the findings. This extensive testing confirmed that TP’s cost-effectiveness is broadly applicable across trauma systems and air medical services, reinforcing confidence that the conclusions are not dependent on specific model assumptions or input values.

###  e) Resuscitation with Prehospital Blood Products Trial (Rephill Trial)

The RePHILL study [31], a multicenter, open-label, phase 3 randomized controlled superiority trial, was conducted across four UK civilian prehospital critical care services. Eligible participants were randomly assigned in a 1:1 ratio to receive either up to 2 units each of packed red blood cells (PRBC) and lyophilized plasma (LyoPlas) or up to 1 L of 0.9% sodium chloride, using identical sealed treatment packs for allocation concealment. The study included adults aged 16 or older with trauma-related hemorrhagic shock and hypotension (systolic blood pressure < 90 mm Hg or absent radial pulse), as assessed by prehospital critical care teams. A total of 432 participants were included in the final analysis.

The primary analysis of the RePHILL study used an intention-to-treat approach, evaluating a composite primary outcome comprising episode mortality, impaired lactate clearance, or both. Of 432 participants randomized (209 to PRBC–LyoPlas and 223 to 0.9% sodium chloride), the composite outcome occurred in 128 (64%) of 199 participants in the PRBC–LyoPlas group and 136 (65%) of 210 in the sodium chloride group. The adjusted risk ratio was 1.01, with an adjusted risk difference of − 0.025%. The trial did not show superiority of prehospital PRBC–LyoPlas over 0.9% sodium chloride in this civilian trauma population, as the wide confidence intervals suggested potential for both benefit and harm.

Sensitivity analyses were conducted to test the robustness of the RePHILL study’s primary outcome, a composite of episode mortality or impaired lactate clearance. These included pre-planned adjustments for baseline factors such as intervention site, cardiac arrest, age, lactate levels, and GCS, as well as analyses addressing lactate timing differences, a per-protocol analysis (including only participants who received assigned interventions), and missing data. *Post hoc* analyses examined subgroups by injury severity and transport time, and simulations modeled outcomes with a target sample size of 490 or up to 5000. All analyses consistently showed no significant difference in the primary outcome between the PRBC–LyoPlas and 0.9% sodium chloride groups, with consistent treatment effects across subgroups. These findings confirmed that prehospital PRBC–LyoPlas was not superior to sodium chloride in this civilian trauma population.

### e) Neural Networks for Trauma Survival Prediction

This study [15] employed a retrospective cohort design to assess predictive models for trauma survival, aiming to enhance audit processes by identifying unexpected outcomes. It compared logistic regression, the foundation of the TRISS methodology, with Multilayer Perceptron (MLP) neural networks, using a dataset of 15,055 cases from Scottish Trauma departments (1992–1996). The dataset was split into a training set (7,224 cases, 1992–1994) and a test set (7,831 cases, 1995–1996) for prospective evaluation. Models were optimized through a two-stage process of backpropagation and quasi-Newton BFGS, using Minimax-scaled inputs and cross-entropy error. Performance was evaluated using Receiver Operating Characteristic (ROC) curves and the area under the curve (AUC). Neural networks outperformed unconditional logistic regression, with the best MLP achieving an ROC AUC of 0.9548, compared to TRISS’s 0.9411. Incorporating patient age directly, rather than TRISS’s binary encoding, significantly improved prediction accuracy across models.

A novel sensitivity analysis was introduced to identify key predictors by replacing each input variable with missing values and measuring the impact on model error, as indicated by the root-mean-square (RMS) of cross-entropy errors. This method, simpler than existing approaches, applied to both numeric and nominal variables and revealed that age, Injury Severity Score (ISS), and Revised Trauma Score (RTS) were the most critical predictors, with age being the most influential. In a detailed model using 14 base variables (components of ISS, RTS, and GCS), variables such as sex, respiratory rate, eye movement, and facial injury showed minimal or negative impact when omitted, suggesting that some TRISS components may be less significant. This analysis supports simplifying scoring schemes by focusing on a smaller set of highly predictive variables, akin to testing a machine’s critical components by removing them one at a time to observe performance changes.

### f) PROMMTT Study

The PROMMTT study [24], a prospective observational multicenter study, evaluated the impact of missing data on three blood transfusion prediction models (McLaughlin, Cancio, and Larson) using 1,245 adult trauma patients (age ≥ 16) from 10 U.S. Level I trauma centers (July 2009–October 2010). Patients arriving directly from the injury scene who received at least one red blood cell (RBC) unit in the Emergency Department (ED) were included, and massive transfusion (MT) was defined as the administration of ≥ 10 RBC units within 24 h. Exclusions included death within 30 min of ED arrival, prior hospital treatment, >5 min of CPR, severe burns, pregnancy, or prisoner status. Clinical data, including injuries, ED vitals, labs, and treatments, were collected prospectively for up to 24 h. However, substantial missing data persisted (e.g., 2.2% for heart rate, 45% for respiratory rate), often higher in MT patients and in those surviving < 6 h, suggesting missingness was either missing at random (MAR) or not at random (MNAR).

The primary analysis compared model performance using complete-case analysis and multiple imputation, finding similar correct classification rates but lower accuracy than in the original validations (e.g., McLaughlin: 60% vs. 70% originally), indicating limited external validity.

A novel sensitivity analysis established upper and lower bounds for classification accuracy by imputing “critical” (95th percentile) and “non-critical” (median) values for MT and non-MT patients, respectively, in best- and worst-case scenarios. Results showed classification ranges of 4% (McLaughlin), 10% (Cancio), and 12% (Larson), with narrower ranges indicating less impact from missing data. This approach highlighted the significant effect of missing data on predictive accuracy in trauma settings, suggesting that reporting these bounds is more informative than relying solely on complete-case analysis or multiple imputation.

### g) PATCH-Trauma Trial

The PATCH-Trauma trial [26], an international, double-blind, randomized, placebo-controlled study, investigated the efficacy and safety of prehospital tranexamic acid (TXA) in 1,310 adult patients (≥ 18 years) with severe traumatic injuries at risk for trauma-induced coagulopathy, treated by paramedics or physicians in Australia, New Zealand, and Germany. Eligible patients had a Coagulopathy of Severe Trauma (COAST) score ≥ 3. They received either TXA or placebo within 3 h of injury before hospital admission, with exclusions for pregnant women and nursing home residents. The primary outcome, assessed via intention-to-treat using log-binomial regression, was survival with a favorable functional outcome at 6 months. Of 661 TXA and 646 placebo patients, 53.7% (307/572) and 53.5% (299/559), respectively, achieved this outcome (risk ratio 1.00), showing no significant TXA benefit. However, TXA showed a trend toward reduced early mortality (e.g., 28-day mortality: 17.3% vs. 21.8%; risk ratio, 0.79). It did not improve long-term functional outcomes, and no significant differences in serious adverse events, including vascular occlusive events, were observed.

To ensure the robustness of these findings, the trial conducted several sensitivity analyses. These included multiple imputations to address missing primary and secondary outcome data, ensuring that incomplete records did not skew results. A per-protocol analysis was also performed, including only patients who received the assigned TXA or placebo intervention as intended, to assess the impact of adherence to the protocol. Additionally, a *post hoc* analysis examined whether the effect of TXA differed between patients with and without TBI, testing for potential subgroup-specific benefits. Further sensitivity analyses explored prespecified subgroups based on variables such as age, mechanism of injury (e.g., blunt vs. penetrating), baseline blood pressure, GCS, and time from injury to first dose, to evaluate consistency of TXA’s effect across diverse patient characteristics. These analyses consistently showed homogeneity in TXA’s impact on the primary outcome, reinforcing the primary finding of no significant improvement in favorable functional outcomes. The lack of variation across subgroups and analytical approaches, combined with the imputation and per-protocol results, provided evidence that the null effect was not an artifact of missing data, protocol deviations, or specific patient characteristics. Thus, the SAs supported the conclusion that TXA does not enhance long-term survival quality in advanced trauma systems despite a potential reduction in early mortality.

###  h) Prehospital Plasma Transfusion Versus Standard of Care Following Traumatic Injury

El-Menyar et al. [29] conducted a meta-analysis to address the effect of Prehospital Plasma Transfusion (PHP) Versus Standard of Care following Traumatic Injury. Seven studies evaluated 24-hour mortality, including 2,170 patients. The overall pooled analysis revealed no significant benefit to PHP transfusion in trauma patients. However, the sensitivity analysis showed a significant association of PHP and lower 24-hour mortality. Heterogeneity was assessed using the I^2^ metric (which reflects the percent variation across studies to true heterogeneity rather than random chance or sampling error), and χ^2^ tests. Sensitivity analysis was performed after excluding small studies with high variance. Despite the pooled effect of PHP use (RCT and non-RCT studies) showing lower 24-hour mortality compared to controls, the difference was not statistically significant [RR 0.92 (95% CI: 0.62–1.38)]. The studies showed moderate heterogeneity (I^2^ = 58.8%). Studies by Kim et al. and Mitra et al. showed high variances due to small sample sizes; therefore, both were excluded from estimation of the effect size. In addition, the study by Holcomb et al. was excluded from the sensitivity analysis because the intervention consisted of plasma and/or RBC. The two small-sample studies had weights of 9% and 4% in the random-effects model. They largely contributed to heterogeneity (I^2^ >50%). After removing these three studies, the heterogeneity dropped considerably (I^2^ = 14%). For the overall analysis, including these three studies, the fixed-effects model showed a significant difference; however, due to heterogeneity, we opted for the random-effects model.

**Fig. 1:**
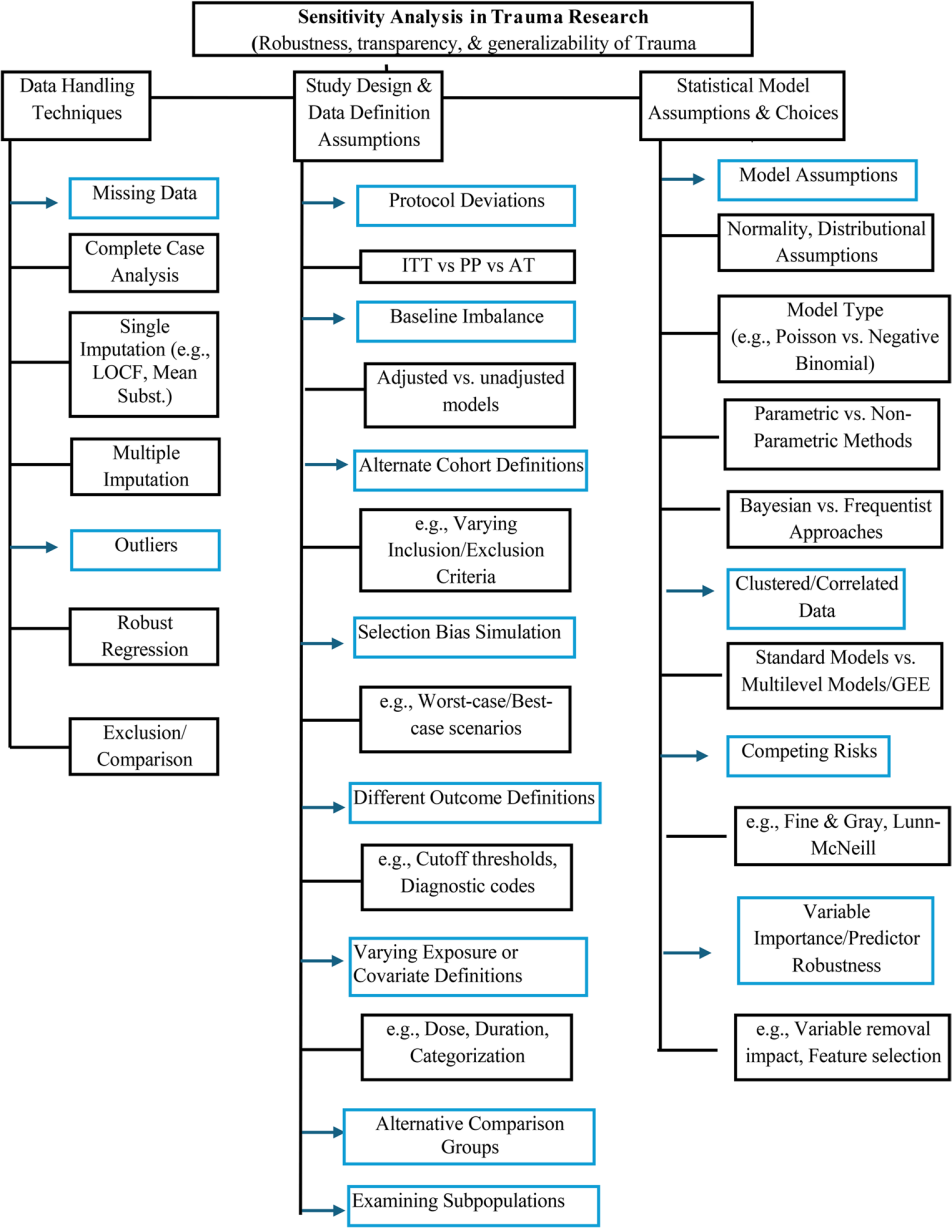
Framework of Sensitivity Analysis Methods Applied in Trauma Research** (**ITT: Intention-to-Treat; PP: Per Protocol, AT: As-Treated)

The examples presented in this section are summarized in Table 1 and collectively illustrate how various sensitivity analysis methods are applied in trauma research. Together, they demonstrate the use of the methodological framework outlined in Fig. 1 to rigorously assess the robustness, reliability, and generalizability of study findings within this complex field.

**Table 1:** Summary of sensitivity analysis (SA) applications and insights in selected studies

Study	Focus of SA	SA Approach	Findings/Implications
Hunter et al. [15]	Survival prediction in trauma patients	To identify the most influential predictors in neural network and TRISS models	Neural networks outperformed TRISS; age, ISS, and RTS were key predictors, enabling the development of simpler scoring systems.
PROMMTT [24]	Massive transfusion (MT) prediction in trauma patients	To assess the impact of missing data on the transfusion model accuracy	Missing data reduced model accuracy; imputation narrowed classification ranges (4–12%), enhancing model reliability.
PROPPR [25]	30-day mortality with transfusion ratios	To evaluate the effect of missing data and protocol deviations on mortality outcomes	A 1:1:1 transfusion ratio was maintained, resulting in a mortality benefit despite missing data, ensuring consistent findings.
PATCH-Trauma [26]	Functional outcome with prehospital tranexamic acid (TXA)	To test the consistency of TXA’s effect across missing data and subgroups	No functional outcome benefit with TXA; consistent null effect across subgroups supported robust conclusions
PAMPer-Trauma Trial [27]	30-day mortality with prehospital plasma	To confirm the robustness of plasma’s survival benefit under varied assumptions	Plasma reduced 30-day mortality consistently, with robust findings supporting its broad applicability.
PAMPer Secondary – Plasma Age [28]	Mortality with plasma storage duration	To compare outcomes between “YOUNG” (0–1 days) and OLD” (3–5 days) plasma groups in transfusion	No mortality differences were observed across plasma ages; consistent efficacy was demonstrated up to 5 days of validated storage practices.
PAMPer Biomarker Study [29]	Biomarker response post-plasma transfusion	To verify the consistency of biomarker effects in high-severity trauma	Biomarker effects were stable in patients with severe trauma; the findings supported the biological mechanism of plasma.
El-Menyar et al. [32]	Prehospital Plasma Transfusion Versus Standard of Care following Traumatic Injury	To distinguish the effects of pRBC combined with plasma versus plasma alone within the intervention group	The two small-sample-size studies had weights of 9% and 4% in the random effect model. They largely contributed to heterogeneity (I^2^ > 50%). After removing these studies, the heterogeneity dropped considerably (I^2^ = 14%).
PAMPer Cost-effectiveness [30]	Cost-effectiveness of prehospital plasma	To evaluate the cost-effectiveness of plasma across varying clinical and system factors	Plasma remained cost-effective in more than 81% of scenarios; the findings endorsed its economic viability.
RePHILL Trial [31]	Mortality and lactate clearance with PRBC + plasma vs. saline	To assess the consistency of the treatment effect across subgroups and data scenarios	No benefit in mortality or lactate clearance; consistent null effect confirmed reliable conclusions

*TRISS* Trauma Score and Injury Severity Score,* ISS* Injury Severity Score, *RTS* Revised Trauma Score, *pRBC* Packed Red Blood Cells, *PROPPR* Pragmatic, Randomized Optimal Platelet and Plasma Ratios trial, *PAMPer* Prehospital Air Medical Plasma trial, *PATCH* Prehospital Antifibrinolytics for Traumatic Coagulopathy and Haemorrhage, *PROMMTT* Prospective Observational Multicenter Major Trauma Transfusion study, *RePHILL* Resuscitation with Prehospital Blood Products

## Challenges and Future Directions for Sensitivity Analysis in Trauma Research

Although SA is essential for enhancing the credibility and interpretability of trauma research, more widespread implementation faces significant challenges. The inherent complexities of trauma studies, including heterogeneous patient populations, variable data quality, and rapidly changing clinical environments, present methodological, practical, and ethical obstacles. Methodologically, selecting appropriate SA techniques that balance complexity with interpretability can be difficult, particularly in the presence of missing data, non-random sampling, or unmeasured confounding. Practically, limited resources, inconsistent data capture, and disintegrated trauma registries hinder the ability to conduct comprehensive SAs. Ethically, ensuring transparency and protecting vulnerable populations requires consideration and attention. Looking ahead, more advanced computational tools, more integration of machine learning/narrow artificial intelligence approaches, and the development of standardized SA frameworks tailored to trauma research offer promising avenues for future exploration. Improved training and capacity-building among trauma researchers will also be vital to advance the effective and appropriate use of SA. These efforts will collectively enhance the robustness and clinical relevance of trauma research findings.

Finally, the evolving nature of SA, especially with the emerging integration of artificial intelligence, means that some emerging techniques and their implications may not yet be fully captured or understood in the current literature.

##  Limitations

While this review offers a comprehensive exploration of sensitivity analysis (SA) in trauma research, there are several limitations. First, this review is primarily narrative and not systematic; as such, it may not capture all relevant studies or SA methodologies in trauma settings. Second, the chosen examples and case studies are predominantly derived from high-income countries and well-resourced trauma systems, potentially limiting the generalizability of findings and best practices to more resource-constrained low- and middle-income countries.

## Conclusion

This review highlights the indispensable role of SA in trauma research, a field marked by substantial patient heterogeneity, complex and dynamic data environments, and challenging ethical considerations. Examples from landmark studies and advanced neural network applications illustrate how SA can enhance the robustness, credibility, and generalizability of scientific findings and help transform them into more actionable insights. Despite its critical value, SA remains underused, hindered by practical and methodological limitations inherent in real-world trauma settings.

## Data Availability

No datasets were generated or analysed during the current study.
